# Serum matrix metalloproteinase 9 and colorectal neoplasia: a community-based evaluation of a potential diagnostic test

**DOI:** 10.1038/bjc.2012.93

**Published:** 2012-03-20

**Authors:** S Wilson, S Damery, D D Stocken, G Dowswell, R Holder, S T Ward, V Redman, M J Wakelam, J James, F D R Hobbs, T Ismail

**Affiliations:** 1 Department of Primary Care Clinical Sciences, University of Birmingham, Edgbaston, Birmingham, B15 2TT, UK; 2 Cancer Research UK Clinical Trials Unit, School of Cancer Sciences, University of Birmingham, Edgbaston, Birmingham, B15 2TT, UK; 3 University Hospitals Birmingham NHS Foundation Trust, Queen Elizabeth Hospital, Mindelsohn Way, Edgbaston, Birmingham, B15 2WB, UK; 4 The Babraham Institute, Babraham Research Campus, Cambridge, CB22 3AT, UK; 5 Cancer Research UK Institute for Cancer Studies, University of Birmingham, Edgbaston, Birmingham, B15 2TT, UK; 6 Department of Primary Care Health Sciences, 2nd Floor, 23-38 Hythe Bridge Street, Oxford, OX1 2ET, UK

**Keywords:** colorectal cancer, matrix metalloproteinase, diagnosis, serum, screening, risk

## Abstract

**Background:**

A blood test may be a more acceptable routine colorectal cancer (CRC) screening test than faecal occult blood test, flexible sigmoidoscopy or colonoscopy, and could be safer and cheaper. We evaluated the accuracy of a serum matrix metalloproteinase (MMP9) test for CRC in a non-presenting symptomatic population.

**Methods:**

A cohort, aged 50–69 with lower gastrointestinal symptoms, was identified by community-based survey. Accuracy of serum MMP9 was assessed by comparison with colonoscopy. Logistic regression identified predictors of neoplasia and receiver operating characteristic curve analyses determined the cutoff to maximise the sensitivity.

**Results:**

Data were available for 748 patients. Overall, 46 cases of neoplasia were identified. Univariate analysis demonstrated that demographic characteristics, behavioural factors, clinical symptoms and raised serum MMP9 concentration were all significantly associated with the presence of neoplasia. Our final logistic regression model had a sensitivity of 79% and specificity of 70%.

**Conclusion:**

We demonstrated a significant association between serum MMP9 concentration and the presence of neoplasia. Serum MMP9 levels are raised in those with cancer and high-risk adenomas, although MMP9 estimation is likely to have the greatest predictive utility when used as part of a panel of biomarkers. Further work is required to identify biomarkers that are sufficiently accurate for implementing into routine practice.

Colorectal cancer (CRC) is a major public health problem (Office for National Statistics, 2010). There are over 37 000 incident cases and over 16 000 deaths in UK each year, incurring an annual expenditure of more than £300 million in surgical, adjuvant and palliative treatment (Macafee *et al*, 2006; Cancer Research UK, 2010a, 2010b). The 5-year age-standardised relative survival rate is improving but is still only around 50% for men and women with cancer of the colon (Cancer Research UK, 2010c). Survival is strongly associated with stage at presentation: the 5-year relative survival rate for people with localised cancer being 90% and the rate for those with distant disease only 12% (National Cancer Institute, 2010). Most CRCs arise from adenomas, and estimates of the progression rate suggest that there is a long window during which screening could facilitate early detection and adenoma removal (Chen *et al*, 2003). Colorectal cancer is, therefore, a suitable candidate for mass screening, and a bowel cancer-screening programme has now been introduced in UK. Screening for 60–69-year olds has commenced throughout England with an extension to include those up to age 74 ongoing (Atkin, 2006; Department of Health, 2007).

The screening programme in the UK uses the guaiac faecal occult blood test (FOBt). Meta-analysis of randomised controlled trials estimates that screening for CRC using FOBt reduces mortality from CRC by 16% (Hewitson *et al*, 2007), but a low positive predictive value (12% for CRC, 46% for all neoplasia) (Hardcastle *et al*, 1996) means that many positive tests are false and subsequent investigations incur unnecessary costs, risks and anxiety. The faecal occult blood test also has a relatively low acceptability, with reported uptake rates in UK of only 55–60% (UK Colorectal Cancer Screening Pilot Group, 2004; Weller *et al*, 2007). A large UK study has assessed whether flexible sigmoidoscopy screening can reduce the incidence and mortality of CRC in 55–64-year olds, and concluded that incidence is reduced by a third and mortality by 43% (Atkin *et al*, 2010). Despite these promising findings, flexible sigmoidoscopy remains costly, invasive and has low acceptability: <50% of people aged 50–75 years accepted an invitation for screening (Verne *et al*, 1998; Atkin *et al*, 2010). Colonoscopy is the most accurate way to detect pathology, but it requires secondary care resources, is unacceptable to some, and has an element of risk. A recent review of 37 studies concluded that the rates of perforation and haemorrhage in colonoscopy are generally <0.1% and <0.3%, respectively, but have been shown to be as high as 0.25% for perforation and 2.1% for bleeding (Juillerat *et al*, 2009, with overview in Arditi *et al*, 2009). The cost, low acceptability and risk make colonoscopy unsuitable as a routine screening test.

A blood test is likely to be more acceptable for routine screening than FOBt, flexible sigmoidoscopy or colonoscopy and could be safer and cheaper. Matrix metalloproteinases (MMPs) are proteolytic enzymes that are associated with tissue remodelling in normal and pathological processes (Collins *et al*, 2001). Overexpression of tissue MMPs has been correlated with progression in many tumour types, and overexpression of MMP9 has been found in colorectal adenomas (Parsons *et al*, 1998) and carcinomas (Garbett *et al*, 1999; Heslin *et al*, 2001). A significant positive correlation has also been found between tissue MMP9 and the stage of colorectal tumours at diagnosis (Baker and Leaper, 2003).

Overexpression of MMP9 in tumours led investigators to question whether measurement of serum levels may provide a useful diagnostic test. Serum MMP9 levels have recently been shown to be significantly elevated in CRC patients compared with controls, and higher in late-stage colorectal neoplasia as compared with early-stage disease (Dragutinovic *et al*, 2011). Our pilot work suggested that serum MMP9 estimation is an acceptable test and has a high sensitivity and negative predictive value for colorectal neoplasia (Hurst *et al*, 2007). Attention has been drawn to the need for robust serum sampling and processing protocols (Jung, 2008), but despite these concerns, serum MMP9 remains a potentially accurate, low-risk and cost-effective population-screening tool. It is, therefore, important to evaluate its accuracy as a test for CRC in the target population within primary care.

This study aimed to evaluate the accuracy of serum MMP9 as a test for CRC in non-presenting symptomatic people in the community.

## Materials and methods

The design of this study is reported in detail elsewhere (Wilson *et al*, 2006). Briefly, a cohort aged 50–69 years with lower gastrointestinal (GI) symptoms was identified via a community-based postal survey. Accuracy of serum MMP9 estimation was assessed by comparison with the gold standard, colonoscopy.

A total of 30 randomly selected general practices in the South Birmingham area were initially invited to participate in the study, of which 19 responded positively to the invitation. Lists of eligible persons were generated from practice registers. General practitioners (GPs) excluded people under investigation or treatment for CRC, unfit for colonoscopy, unable to give informed consent, or who should not be invited to participate for any other reason (for example, recent bereavement or terminal illness). A self-administered questionnaire has been shown to be a useful tool to assess bowel symptoms with a reasonable agreement with symptoms reported from a patient–doctor interaction (Adelstein *et al*, 2008). A postal questionnaire assessed potential GI alarm symptoms used in the National Institute for Health and Clinical Excellence (NICE) guidelines for referral of suspected CRC (National Institute for Health and Clinical Excellence (NICE), 2005), the relative acceptability of screening tests for CRC, risk factors for CRC, and willingness to take part in the study. One reminder was sent. Respondents who described any colorectal symptoms (except only abdominal bloating and/or anal symptoms) and indicated their willingness to take part in the study were sent an information leaflet and an invitation to attend a research clinic at their general practice. At the general practice clinic, the study was described, fitness for colonoscopy assessed, a basic medical history obtained and informed consent sought.

Colonoscopies were performed in a dedicated research endoscopy unit based on the Wellcome Trust Clinical Research Facility, Queen Elizabeth Hospital, Birmingham. Colonoscopy findings were categorised as adenocarcinoma, high-risk or low-risk polyp, benign inflammatory disease of the colon, other benign disease of the colon, other benign conditions and no abnormality; the first two categories (adenocarcinoma and high-risk adenoma) being classified as neoplasia for the purposes of the analysis (Atkin and Saunders, 2002).

Two 5 ml blood samples were collected immediately before colonoscopy: one for routine analysis (haemoglobin, erythrocyte sedimentation rate, mean corpuscular volume, white blood cell count) in a purple-top tube containing EDTA (Vacuette, Greiner, Kremsmûnster, Austria), the other for MMP9 determination in a red-top tube with no additive. Samples were kept on ice until the end of the colonoscopy list at which point they were delivered to the laboratory, immediately centrifuged and the serum fraction stored at −80 °C. MMP9 levels were determined on duplicate aliquots of each sample by ELISA assay using a commercially available kit (R&D Systems, Abingdon, UK). All blood samples were analysed in the same laboratory to ensure standardisation of measurement and reporting. The technician undertaking the MMP9 ELISA assay was blinded to each patient's self-reported symptoms. All participants were flagged on the NHS Central Register to maximise the ascertainment of malignancy in those with no abnormality detected during colonoscopy.

### Justification of sample size

Any test that might usefully be employed as a symptomatic screen for malignant or premalignant colorectal lesions should have a high specificity to minimise the number of non-malignancies incorrectly identified as malignant. On the basis of our pilot data and conservatively assuming a community prevalence of neoplasia of 6% (Thiis-Evensen *et al*, 1999; Lieberman *et al*, 2000), a sample of 700 people would be sufficient to estimate specificity within 4% (95% confidence). If the community prevalence of neoplasia was >6%, our estimate of specificity would have greater precision.

On the basis of our pilot data (3.2% of the age group being suitable and consenting) and assuming an average practice list of 4500 with 18% of people aged 50–69 (Office for National Statistics, 2004), 23 100 people from 29 practices were estimated to be required to identify 700 eligible participants consenting to colonoscopy and MMP9 determination. In the study itself, the required number of patients was reached from a lower number of practices than initially anticipated (*n*=19), due to a larger than expected average list size (9000) in participating practices.

### Methods of data analysis

Data were checked for completeness and accuracy. Outliers were identified and validity was assured. Patients were classified into two pre-specified diagnostic groups; ‘neoplasia’ group: patients diagnosed with adenocarcinoma or high-risk polyps, and ‘non-neoplasia’ group: low-risk polyps, benign tumours or no abnormality.

Continuous data, including MMP9 measurements, were not normally distributed and are presented as medians and inter-quartile ranges. Univariate comparisons used the non-parametric Mann–Whitney *U* test. Categorical measurements are presented as proportions and compared using *χ*^2^ tests. Odds ratios, adjusted for all other predictive variables, were derived from logistic regression modelling to ‘predict’ patients with neoplasia. All appropriate factors (demographics, haematology, reported symptoms and pre-existing medical conditions) were considered in the logistic regression analyses. Actual serum MMP9 concentrations were initially used but an additional variable was created using quartiles (<144, 144 to <260, 260 to <424, 424+ ng ml^−1^). Each continuous variable was assessed for the assumption of linearity with outcome and appropriate transformations applied where applicable. Logistic regression analyses were based on a complete case analysis using variables (initially on their own), which were significant at or near the 10% level. This was to ensure that clinical relevance in the model was not sacrificed for parsimony. A novel two-stage logistic regression approach was used to exploit the imbalance between the number of positive and negative neoplasia patients. Taking a cutoff point of 0.05 on the predicted probability of neoplasia from the logistic regression model, all patients predicted as positive from this process were re-entered into a second logistic regression, where a cutoff of 0.05 was also taken as the most appropriate. Combining the results of this two-stage process produced a more successful predictive procedure.

Model accuracy was calculated with the aim of achieving a balance of sensitivity and specificity. Receiver operating characteristic curves were plotted, recording sensitivity *vs* 1-specificity for all probability cut-points of the predictive model. The probability cut-point for prediction of neoplasia for specified levels of sensitivity and specificity can be identified and was chosen to maximise sensitivity as indicated in the study protocol.

## Results

### Response rates

**Figure 1:**
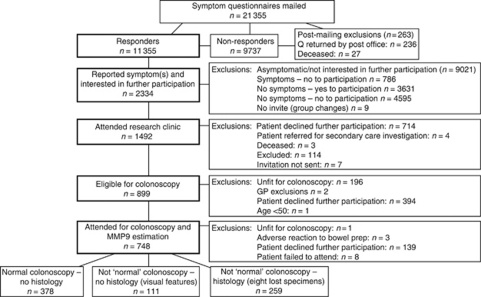
Study Profile.

Searches of GP registers identified 21 488 patients aged 50–69, of which 133 (0.6%) were excluded by their GP and 21 355 sent a screening questionnaire (willingness to participate further and symptom profiles), (Figure 1). Responses were received from 53% of those contacted (*n*=11 355). Colorectal symptoms (except only abdominal bloating and/or anal symptoms) were reported by 27.5% of responders (*n*=3124), and 2334 (20.6%) reported willingness to take part in the evaluation of MMP9. After attendance at a research clinic, to obtain consent and evaluate fitness for colonoscopy, 751 patients were identified as being eligible for further participation, of which 748 had a colonoscopy and provided a serum sample for MMP9 determination.

### Sample characteristics

Slightly lower participation rates were observed among males (*χ*^2^=122.8, *P*=<0.001), younger people (*t*=7.607, *P*=<0.001) and in those from the most affluent areas (*χ*^2^=20.64, *P*⩽0.001). However, despite these differential response rates, the final study population was similar to that of the West Midlands region and England as a whole (Office for National Statistics, 2005) with respect to gender (proportion male: 47.6% (study) *vs* 49.5% in West Midlands and 49.1% in England), age distribution (proportion of study age-group, i.e. 50–69, aged 60–69 years: 41.2% (study) *vs* 44.5% (West Midlands) *vs* 43.4% (England)), and deprivation score (very affluent: 13.9% (study) *vs* 19.4% (West Midlands) *vs* 25.0% (England)).

The median age of participants was 59 years (IQR: 54–63), 47.6% (*n*=356) were male, 92.0% (*n*=688) white, and 25.8% (*n*=193) lived in areas classified as socioeconomically deprived (Table 1). The most commonly reported symptoms were anal pain (53.0% median duration of symptoms: 12 weeks; IQR: 3–30 weeks), change in bowel habit to looser stools (45.2% median duration of symptoms: 10 weeks; IQR: 2.3–26), tiredness (43.7% median duration: 12 weeks; IQR: 6–26), increased bowel frequency (39.0% median duration: 10 weeks; IQR: 3–24), rectal bleeding (38.2% median duration: 6 weeks; IQR: 1–30) and change in bowel consistency to harder stools (33.7% median duration: 7 weeks; IQR: 2.8–26). Self-reported symptoms and symptom duration fulfilled the NICE guidelines for 2 week referral for 11.2% (*n*=84) of participants (NICE, 2005).

### Colonoscopy findings

Overall, 46 cases of neoplasia (three colorectal adenocarcinomas and 43 high-risk polyps) were identified on colonoscopy; representing 6% of the study population. Other findings included low-risk polyps (*n*=165) and inflammatory disease (*n*=12).

### Serum MMP9 results

The median serum MMP9 concentration was 260.3 ng ml^−1^; range (35.7–2133.4; IQR: 144.2–423.9) (Table 1). Crude median MMP9 concentration differed between diagnostic groups, with higher MMP9 levels observed in the neoplasia group than the non-neoplasia group. Categorisation of MMP9 levels into quartiles also demonstrated an association between higher MMP9 levels and neoplasia (*χ*^2^=16.7, *P*=0.001) (Table 2).

### Factors associated with the presence of neoplasia

**Figure 2:**
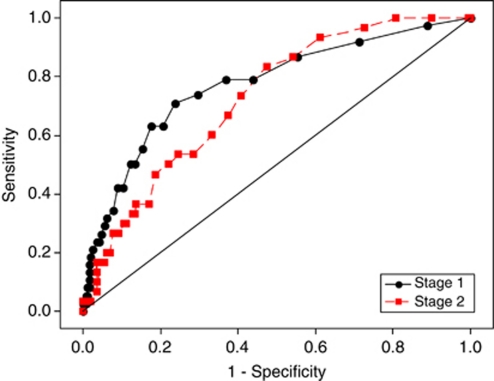
Receiver operating characteristic curves (ROCs) for two-stage binary logistic regression area under curve=0.77 (stage 1) and 0.73 (stage 2).

Univariate analyses, corroborated by a review of the relevant clinical literature, demonstrated that increasing age, male gender, weight loss, reporting blood in the stools, the absence of anal pain, changes in bowel habit to harder stools, smoking, drinking alcohol, raised blood pressure, raised white blood count and raised serum MMP9 concentration were all significantly associated with the presence of neoplasia (Table 2). Entering these individual neoplasia predictive factors into a logistic regression model (Table 3), along with potential confounding factors such as recent injuries, arthritis, muscular problems (which may have affected observed MMP9 measurements) and bearing in mind the expected prevalence of 6%, a cutoff on the predicted probability of neoplasia of 0.05 (5%) resulted in a sensitivity of 79% and specificity of 63%. The 249 patients predicted by the model to be in the neoplasia group were then entered into a further logistic regression, (Table 3) and results from the two stages were combined (Table 4). Receiver operating characteristic curves are also presented for the combined logistic regression (Figure 2). Using a cutoff of 0.05 in the two stage model, sensitivity was maintained at 79% but specificity was improved to 70%. Advanced statistical analysis (neural networks, recursive partitions and regression trees) did not give any improvement in model performance.

## Discussion

This study investigated the association between MMP9 and colorectal neoplasia in a non-presenting cohort of patients selected on the basis of prompted self-reported symptoms. We have demonstrated a significant association between serum MMP9 concentration and the presence of neoplasia and this study supports previous work, which reported that patients referred with suspected neoplasia had significantly elevated MMP9 levels (Hurst *et al*, 2007).

Strengths of this study include duplicate determination of serum MMP9 levels and dual data entry to ensure reliability of results. This study has confirmed that MMP9 levels are raised in those with neoplasia, but a more detailed exploration has also identified that this relationship is non-linear. Categorisation of MMP9 concentrations into four groups enabled the predictive value of MMP9 to be modelled more accurately, given that linearity is not a statistical assumption when categorised data are used (unlike the assumptions limiting the analysis of continuous variables).

A potential limitation of our approach is the use of a non-validated questionnaire to assess patient symptoms. This was unavoidable, as no validated measures existed at the time of data collection for this study. However, the questionnaire was based on the NICE urgent referral guidelines for suspected CRC, and its robustness as a data collection tool was established during pilot work. A further limitation is that patient eligibility for study participation was determined with reference to self-reported symptoms (rather than those assessed by a physician). However, other work has shown that there is a good level of agreement between self-reported and physician-assessed symptoms (Adelstein *et al*, 2008). As an additional safeguard, an algorithm was incorporated into the study database so that the relevant GP could be informed if any of their patients reported symptoms that met the NICE urgent referral criteria, so that clinical investigations could be carried out if necessary.

The use of serum MMP9 estimation has been previously criticised owing to its increased level compared with plasma estimation (Jung, 2008). Matrix metalloproteinase 9 is known to be secreted by platelets and leucocytes when activated by coagulation and fibrinolytic pathways and this effect leads to the elevated levels in serum *vs* plasma. Some blood-sampling tubes used for serum estimations contain clot activators, which have been shown to result in a 15-fold increase in serum MMP9 levels compared with that of citrate plasma (Jung *et al*, 2008). This study used blood sampling tubes that did not contain any clot activators. The time elapsed between blood sampling and centrifugation is associated with higher serum MMP9 levels, with a suggested seven-fold increase after 2 h (Gerlach *et al*, 2007), as demonstrated for a sample left at room temperature for 2 h rather than kept on ice, as in our study. Reassuringly, our data showed no correlation between time to centrifugation and serum MMP9 level (Pearson correlation, *r*=−0.010, *P*=0.801).

One study has measured both serum and plasma MMP9 levels in patients with gastric cancer and controls (Wu *et al*, 2007). This study demonstrated a significant difference between plasma MMP9 levels in cancer patients compared with controls but no such difference for serum levels. Robust methods for serum collection and processing were not described and this could have explained the lack of demonstrated association.

Despite citrate plasma being the suggested sample of choice for estimating circulating MMP9 (Makowski and Ramsby, 2003), serum sampling may still be useful providing that methods of collection and processing are standardised. Serum levels have thus been shown to correlate significantly with plasma levels in two different diseases (Gerlach *et al*, 2009). Serum MMP9 levels have also been shown to be significantly associated with breast cancer stage and size (Motovali-Bashi *et al*, 2010), gastric cancer stage (Dragutinovic *et al*, 2009), stromal reaction in gastric cancer (Shen *et al*, 2000), and CRC stage (Dragutinovic *et al*, 2011).

The current study supports our pilot work (Hurst *et al*, 2007) in demonstrating that relative levels of serum MMP9 concentration may have some potential in the prediction of significant colorectal pathology. The pilot work, which assessed 300 urgent referrals to colorectal outpatients, demonstrated a difference in median MMP9 concentrations between non-neoplastic and neoplastic groups of 443 ng ml^−1^, The current study, with a larger population, suggests a much smaller difference between the groups (153 ng ml^−1^). Despite our predictive model having a reasonable sensitivity and specificity, MMP9 alone does not appear sufficiently accurate to be usefully incorporated into clinical practice. Our model, incorporating MMP9, age, gender, weight loss, blood in the stool, harder stools, anal pain, white blood count, smoking and alcohol, achieved a sensitivity of 79% and a specificity of 70%, yet this sensitivity and specificity was achievable only via a relatively complex two-stage regression model. The statistical complexity of this model prohibits its routine implementation into clinical practice.

An accurate biomarker for CRC would have potential as an alternative screening method to FOBt (which has relatively low acceptability), or as an aid to GPs in determining which patients required urgent referral for suspected CRC. Nevertheless, the size of the effect demonstrated by this study is insufficient for us to be able to suggest that serum MMP9 is such a biomarker. Ongoing work will aim to determine whether a panel of biomarkers would be sufficiently accurate to have a useful role as a screening test or to aid referral decisions. Typically, biomarker discovery studies are undertaken on selected groups of patients due to the cost and time involved in recruiting a representative sample of the population. We sent screening questionnaires to 21 355 patients aged 50–69, registered with 19 general practices and this study took 3 years to complete. Our results demonstrate the need for the collection and maintenance of serum and plasma banks to validate the accuracy of putative biomarkers.

## Figures and Tables

**Table 1:** Patient characteristics

	**Number (%)^a^**
**Age, median=59 years**	**Range=50–70 years^b^ (IQR: 54–63)**
*Demographics (*n=*748 patients)*	
Gender	
Male	356 (47.6)
Female	392 (52.4)
	
Ethnic group	
White	688 (92.0)
Non-white	60 (8.0)
	
Deprivation quartile (IMD 2004)	
Quartile 1 (least deprived)	104 (13.9)
Quartile 2	187 (25.0)
Quartile 3	263 (35.2)
Quartile 4 (most deprived)	193 (25.8)
	
BMI^c^	
Median (*n*=677; BMI: 27.1)	Range**=**17.2–59.7 (IQR: 24.7–30.4)
	
Drink	
Yes	562 (75.1)
No	185 (24.7)
	
Smoke	
Yes	117 (15.6)
No	631 (84.4)
	
Still menstruating (females only)	
Yes	30 (7.7)
No	362 (92.3)

**Symptom**	**Yes (%)**	**No (%)**	**Missing (%)**	**Duration (IQR)^d^**
*Reported symptoms, median duration in weeks*				
Abdominal pain	235 (31.4)	469 (62.7)	44 (5.9)	4 (2–20)
Weight loss	29 (3.9)	686 (91.7)	33 (4.4)	12 (9–31)
Tiredness	327 (43.7)	377 (50.4)	44 (5.9)	12 (6–26)
Blood in stools	97 (13.0)	602 (80.5)	49 (6.6)	11 (2–57)
Bleeding from back passage	286 (38.2)	435 (58.2)	27 (3.6)	6 (1–30)
Bowel habit–harder stools	252 (33.7)	450 (60.2)	46 (6.1)	7 (2.8–26)
Bowel habit–looser stools	338 (45.2)	357 (47.7)	53 (7.1)	10 (2.3–26)
Open bowels less frequently	155 (20.7)	533 (70.3)	60 (8.0)	10 (4–26)
Open bowels more frequently	290 (39.0)	404 (54.0)	54 (7.2)	10 (3–24)
Anal pain	397 (53.0)	331 (44.3)	20 (2.7)	12 (3–30)
Referral group^e^	Urgent referral: *n*=84, 11.2%			
	Non-urgent referral; *n*=664, 88.8%			

**Condition**	**Yes (%)**	**No (%)**	**Missing (%)**
*Medical conditions*			
Heart disease	38 (5.1)	707 (94.1)	3 (0.8)
Gastrointestinal surgery	20 (2.7)	727 (97.2)	1 (0.1)
High blood pressure	194 (25.9)	553 (74.0)	1 (0.1)
Diabetes	35 (4.7)	711 (95.1)	2 (0.2)
Kidney disease	9 (1.2)	738 (98.7)	1 (0.1)
Respiratory problems	102 (13.6)	643 (86.0)	3 (0.4)
Irritable bowel disease	4 (0.5)	743 (99.4)	1 (0.1)
Arthritis	292 (39.0)	454 (60.7)	2 (0.2)
Muscular problems	61 (8.2)	686 (91.7)	1 (0.1)
Epilepsy	7 (10.0)	740 (98.9)	1 (0.1)
Other medical condition	328 (43.9)	420 (56.1)	0 (0.0)
Injuries <3 months before	37 (4.9)	710 (95.0)	1 (0.1)
Previous bowel polyps	25 (3.3)	723 (96.7)	0 (0.0)
Previous bowel cancer	0 (0.0)	747 (99.9)	1 (0.1)
Family history of bowel cancer	184 (24.6)	563 (75.3)	1 (0.1)

**Measurement**	**Number (%)**	**Missing (%)**	**Median**	**Range (IQR)**
*Haematology and MMP9 measurements*				
ESR	688 (92.0)	60 (8.0)	8.0	0–56 (5.0–13.0)
MCV	722 (96.5)	26 (3.5)	88.2	56.8–101.2 (85.7–90.9)
Hb	722 (96.5)	26 (3.5)	14.5	7.7–18.1 (13.6–15.4)
WBC	722 (96.5)	26 (3.5)	6.3	2.4–20.9 (5.2–7.3)
MMP9 (crude)	748 (100.0)	0 (0.0)	260.3	35.7–2133.4 (144.2–423.9)

Abbreviations: BMI=body mass index; ESR=erythrocyte sedimentation rate; Hb=haemoglobin; IMD=index of multiple deprivation; IQR=inter-quartile range; MCV=mean cell volume; MMP=metalloproteinase; WBC=white blood count.

a Percentages may not total 100% for all demographic variables due to missing data.

b Although the study population was aged between 50 and 69 at the time of recruitment, delays between study enrolment and colonoscopy meant that two patients had turned 70 in the intervening period.

c Body mass index (BMI) was calculated for each individual using self-reported height and weight.

d Figures relating to the duration of reported symptoms are measured in weeks.

e Referral group denotes the proportion of patients who met the NICE urgent referral criteria for suspected colorectal cancer following their reported symptoms.

**Table 2:** Factors considered predictive of neoplasia

			**Neoplasia**	**Non-neoplasia**		
**Variable**	***N***		**Median (IQR)**	**Median (IQR)**	***U***	***P*-value**
Age	748		60 (56–65)	58 (54–63)	13171	0.036
WBC	722		6.7 (5.6–7.8)	6.2 (5.2–7.4)	11900	0.046
MMP9	748		407 (168–655)	254 (142–410)	12529	0.003
					
**Variable**	**Category**	***n***	***n* (Row %)**	***n* (Row %)**	***χ* ^2^**	***P*-value**
Gender	Male	356	28 (7.9)	328 (92.1)	3.46	0.063
	Female	392	18 (4.6)	374 (95.4)		
Weight loss	Yes	29	4 (13.8)	25 (86.2)	3.05	0.081
	No	686	40 (5.8)	646 (94.2)		
	Missing	33	2 (6.0)	31 (94.0)		
Blood in stools	Yes	97	12 (12.4)	85 (87.6)	7.55	0.006
	No	602	31 (5.1)	571 (94.9)		
	Missing	49	3 (6.1)	46 (93.9)		
Harder stools	Yes	252	11 (4.4)	241 (95.6)	2.42	0.12
	No	450	33 (7.4)	417 (92.7)		
	Missing	46	2 (4.3)	44 (95.7)		
Anal pain	Yes	397	18 (4.5)	379 (95.5)	4.70	0.030
	No	331	28 (8.5)	303 (91.5)		
	Missing	20	0 (0.0)	20 (100.0)		
Smoke	Yes	117	11 (9.4)	106 (90.6)	3.07	0.08
	No	631	33 (5.5)	596 (94.5)		
Drink	Yes	562	39 (6.9)	523 (93.1)	2.40	0.121
	No	185	7 (3.8)	178 (96.2)		
	Missing	1	0 (0.0)	1 (100.0)		
High blood pressure	Yes	194	18 (8.8)	177 (91.2)	3.08	0.079
	No	553	29 (5.2)	524 (94.8)		
	Missing	1	0 (0.0)	1 (100.0)		
MMP9 quartiles	0 (lowest)	187	6 (3.2)	181 (96.8)	16.7	0.001
	1	187	8 (4.3)	179 (95.7)		
	2	187	9 (4.8)	178 (95.2)		
	3 (highest)	187	23 (12.3)	164 (87.7)		

Abbreviations: IQR=inter-quartile range; MMP=metalloproteinase; WBC=white blood count.

**Table 3:** (a) Binary logistic regression to predict neoplasia, (b) All positive cases from the logistic regression in Table 3a entered into a second-stage binary logistic regression

						**95% CI**	
**Predictor**	**Coefficient**	**SE coefficient**	***Z***	***P***	**Odds ratio**	**Lower**	**Upper**
*(a)* a							
Constant	−3.04179	3.18681	−0.95	0.34			
Age	0.0788488	0.0345323	2.28	0.022	1.08	1.01	1.16
Sex	−0.471409	0.374236	−1.26	0.208	0.62	0.3	1.3
Weight loss	−0.662248	0.717769	−0.92	0.356	0.52	0.13	2.11
Blood in stools	−1.29532	0.431269	−3	0.003	0.27	0.12	0.64
Harder stools	0.323738	0.420194	0.77	0.441	1.38	0.61	3.15
Pain/soreness	0.854463	0.372727	2.29	0.022	2.35	1.13	4.88
White blood cell count	0.006843	0.10783	0.06	0.949	1.01	0.82	1.24
Smoke	−0.677386	0.461142	−1.47	0.142	0.51	0.21	1.25
Drink	−0.460766	0.477719	−0.96	0.335	0.63	0.25	1.61
High blood pressure	−0.468897	0.384473	−1.22	0.223	0.63	0.29	1.33
							
*MMP9 quartiles*							
1	1.09352	0.407497	2.68	0.007	2.98	1.34	6.63
2	1.07552	0.545041	1.97	0.048	2.93	1.01	8.53
3	1.00486	0.947643	1.06	0.289	2.73	0.43	17.5
							
*(b)* b							
Constant	−3.62048	3.98767	−0.91	0.364			
Age	0.063214	0.0459165	1.38	0.169	1.07	0.97	1.17
Sex	−0.504719	0.454434	−1.11	0.267	0.6	0.25	1.47
Weight loss	−0.0813844	0.886543	−0.09	0.927	0.92	0.16	5.24
Blood in stools	−1.33046	0.532307	−2.5	0.012	0.26	0.09	0.75
Harder stools	−0.0095251	0.520657	−0.02	0.985	0.99	0.36	2.75
Pain/soreness	1.34709	0.528484	2.55	0.011	3.85	1.37	10.84
White blood cell count	0.0491577	0.11819	0.42	0.677	1.05	0.83	1.32
Smoke	−0.826087	0.515073	−1.6	0.109	0.44	0.16	1.2
Drink	−0.0739359	0.553835	−0.13	0.894	0.93	0.31	2.75
High blood pressure	−0.671765	0.442246	−1.52	0.129	0.51	0.21	1.22
							
*MMP9 quartiles*							
1	1.26914	0.581586	2.18	0.029	3.56	1.14	11.12
2	1.22008	0.672376	1.81	0.07	3.39	0.91	12.65
3	0.879027	1.10707	0.79	0.427	2.41	0.28	21.09

a Log-likelihood=−124.874; test that all slopes are zero: G=37.579, DF=13, *P*-value=0.000.

b Log-likelihood=−83.666; test that all slopes are zero: G=15.875, DF=13, *P*-value=0.256.

**Table 4:** Summary of sensitivity and specificity of binary logistic regression models

**First-stage binary logistic regression: summary (sensitivity=79%, specificity=63%)**			
	**Predicted negative**	**Predicted positive**	**Total**
Non-neoplasia	375	219	594
Neoplasia	8	30	38
Total	383	249	632
			
**Second-stage binary logistic regression (all predicted positive cases re-entered): summary**			
	**Predicted negative**	**Predicted positive**	**Total**
Non-neoplasia	42	177	219
Neoplasia	0	30	30
Total	42	207	249
			
**Combined results from both stages (sensitivity=79%, specificity=70%)**			
**Actual**	**Not predicted**	**Predicted**	**Total**
Non-neoplasia	375+42	177	594
Neoplasia	8+0	30	38
